# Determinants of immunization in polio super high-risk union councils of Pakistan

**DOI:** 10.1016/j.vaccine.2023.12.056

**Published:** 2024-01-25

**Authors:** Ahmad Khan, Imtiaz Hussain, Dale A. Rhoda, Muhammad Umer, Uzair Ansari, Imran Ahmed, Caitlin Clary, Rana Muhammad Safdar, Sajid Bashir Soofi

**Affiliations:** a Center of Excellence in Women & Child Health, The Aga Khan University, Pakistan; b Biostat Global Consulting, Worthington, OH, USA; c Ministry of Health Services Regulation & Coordination, Islamabad, Pakistan; d Department of Pediatrics & Child Health, The Aga Khan University, Pakistan

**Keywords:** Immunization coverage, Fully vaccinated, Polio super high-risk union councils, Pakistan

## Abstract

**Background:**

The current polio epidemiology in Pakistan poses a unique challenge for global eradication as the country is affected by ongoing endemic poliovirus transmission. Across the country, 40 union councils (UCs) which serve as core reservoirs for poliovirus with continuous incidences of polio cases are categorized as super-high-risk union councils (SHRUCs).

**Methodology:**

A cross-sectional survey was conducted in 39 SHRUCs using a two-stage stratified cluster sampling technique. 6,976 children aged 12–23 months were covered. A structured questionnaire was used for data collection. Data were analyzed using STATA version 17.

**Results:**

Based on both vaccination records and recall, 48.3% of children were fully-, 35.4 % were partially-, and 16.3% were non-vaccinated in the SHRUC districts. A child is considered fully vaccinated when h/she completed vaccination for BCG, OPV0, OPV 1-3, Penta 1-3, PCV 1-3, IPV, and MCV1. Vaccination cards were seen for over half of the children in the SHRUC districts of Khyber Pakhtunkhwa (KP) and the majority of the SHRUC districts in Sindh, except for the SHRUC district of Malir the districts of Balochistan.

Results for polio vacancies show that 60.9% of children from the SHRUC districts were vaccinated with at least three doses of OPV and one dose of IPV, while 20.4% were vaccinated with any OPV doses or IPV and 18.7% of children did not receive any polio vaccines. The dropout rate between vaccine visits was higher than the WHO-recommended cutoff point of 10% for all vaccine doses in the SHRUC districts. The likelihood of being fully vaccinated was higher among the children of educated parents. Full vaccination was found significant among the children of any SHRUC districts compared to district Killa Abdullah.

**Conclusion:**

Context-specific strategies with more focus on community engagement and targeted mobilization, along with robust monitoring mechanisms, would help address the underlying challenges of under-immunization in the SHRUCs.

## Highlights

• 48.3% children are fully vaccinated in the super-high-risk union councils districts of Pakistan.
• Vaccination coverage varies considerably across the super-high-risk union council districts.
• Dropout rate between vaccine visits is as higher as 60.5% and as low as 4.9% in the districts.
• Full immunization is associated with parental education level.

## 1 Background

Immunization is one of the most cost-effective and proven public health interventions available to avert vaccine-preventable diseases (VPDs). It saves over 3.5–5 million lives globally [1]. Nonetheless, despite remarkable progress over the years, global immunization coverage for the third dose of the Pentavalent vaccine (Diphtheria, Tetanus, and Pertussis (DTaP-3), Hib and Hepatitis B) dropped from 86 % in 2019 to 83 % in 2022, leaving 20.5 million children under one year of age unimmunized, while 1.5 million deaths are still occurring each year globally due to the VPDs [2]. Moreover, infant and child mortality and the number of unimmunized children are persistently high in developing countries [3].

Though Pakistan has made considerable progress in immunization coverage, the immunization indicators have not reached the optimal level. According to the Pakistan Demographic and Health Survey (PDHS) 2018, only 66 % of children aged 12–23 months were fully immunized. Nonetheless, there is encouraging progress as indicated by a national-scale immunization coverage survey in 2020, which reported the national full immunization coverage (FIC) rate at 76.5 % [4], [5].

The key goals of polio eradication, and measles, have not yet been achieved [6] as the country experiences continued incidences of endemic polio transmission and periodic measles outbreaks. Despite continuous efforts, the current polio epidemiology in Pakistan poses a unique challenge for global eradication as the country is affected by ongoing endemic Wild Poliovirus type 1 (WPV1) transmission and circulating vaccine-derived poliovirus type 2 (cVDPV2). In 2020, 84 WPV1 and 135 cVDPV2 cases were reported in Pakistan [7]. However, significant progress was witnessed in 2021 when a single case of WPV1 and eight cases of cVDPV2 were reported in the country. The increasing trend of polio cases continued in 2022, with 19 WPV1 cases surfacing but no cases of cVDPV2 were reported [4]. As of November 2023, the situation appears more promising, with only four cases of WPV1 reported and no instances of cVDPV2 [7]. With these remaining cases, Pakistan is still one of the two polio-endemic countries in the world - a position it holds alongside neighboring Afghanistan [8].

In Pakistan, the coverage estimates at the district and Union Council (UC) level are absent from national demographic and health surveys. The national-level surveys do not provide district and union council-specific estimates for immunization coverage. Across the country, there are 7,387 UCs. Among them, 40 UCs serve as core reservoirs for poliovirus, with continuous incidences of polio cases every year (figure S1). These UCs have been identified as Super-High Risk Union Councils (SHRUCs) by the National Emergency Operations Center (NEOC) for polio eradication in 2019 [9], [10]. The number of SRUCs is contingent on the ongoing circulation of WPV1 and a repeated history of reseeding the virus outside the immediate transmission zone [10]. Since 2019, the number of SHRUCs has remained unchanged, and the list continues to include the same UCs. These SHRUCs have their unique challenges regarding immunization coverage that remain largely under-explored. In the absence of reliable estimates for immunization coverage at the UC level, it is not possible to set targets or measure coverage accurately to inform future policy decisions. Therefore, the Aga Khan University (AKU), with the support of EPI Pakistan, conducted a district-specific Third-party Verification Immunization Coverage Survey (TPVICS) in the country from September 2020 to January 2021 [11]. The survey was meant to assess the coverage and progress of immunization rates nationally to make future policy decisions more evidence-informed. After reviewing the results of the TPVICS [11], the National Immunization Program Pakistan and the key partners supporting the National Immunization Support Project (NISP), decided to conduct a supplementary immunization coverage survey targeting the 39 Polio-SHRUCs in the country [12]. The Aga Khan University (AKU) carried out the supplementary survey to assess and estimate immunization coverage precisely and create valid baseline information for the SHRUCs to assess the impact of future immunization-related interventions.

This paper presents the results of the supplementary immunization coverage survey [12] and also assesses immunization status among the target children to identify UCs with poor coverage.

## 2 Methods

### 2.1 Study design and setting

A cross-sectional survey was conducted in 39 SHRUCs from eight districts in three provinces (Sindh, Khyber Pakhtunkhwa (KP), and Balochistan). The population of the SHRUCs is estimated to be 3,040,205 with 547, 237 children under five years of age [10]. The survey was conducted from July 2021 to October 2021. Children born between July 2019 to October 2019 were enrolled in the survey.

### 2.2 Sampling procedure

A two-stage stratified cluster sampling technique was employed to carry out the survey. The sampling technique adapted from World Health Organization (WHO) recommended sampling methods for immunization coverage cluster survey [13]. In the first stage, the required number of the Primary Sampling Units (PSUs) from each SHRUC were selected randomly with necessary identification information and boundary demarcations using the maps developed and finalized during the provincial workshops organized by Bill and Melinda Gates Foundation (BMGF) for the operationalization of Essential Immunization (EI) work plans in the SHRUCs. In the second stage, eligible Households (HHs) – households with children between 12 and 23 months were treated as Secondary Sampling Units (SSUs) and selected randomly using the new household listing.

Samples from all PSUs/clusters were aggregated at the UC level, and analysis was conducted on UCs and then on upper administrative levels i.e. District level. It is pertinent to mention that SHRUCs constitute a subset of these districts, so the SHRUCs results are not meant to represent the entire district.

### 2.3 Sample size determination

The sample size was estimated by considering a 95 % confidence interval, ±7-8 % level of precision, and 5 % design effect. As a result, a sample of 13 children aged 12–23 months per cluster from each district was required to fulfill the survey goal. The survey covered 610 clusters, 7,549 households, 6,976 children aged 12–23 months, and their mothers/caregivers. Table 2 provides district-wise coverage of samples.

**Table 1:** Expanded Programme on Immunization schedule for children in Pakistan.

**Age**	**When**	**Vaccines**
At Birth	At Birth	BCG, OPV0, HepB
2nd Visit	6 Weeks	OPV1, Rotavirus1, PCV1, Pentavalent1
3rd Visit	10 Weeks	OPV2, Rotavirus2, PCV2, Pentavalent2
4th visit	14 Weeks	OPV3, IPV1, PCV3, Pentavalent3
5th Visit	9 Months	Measles1, IPV2*, Typhoid*
6th Visit	15 Months	Measles2

Abbreviations: BCG, bacillus calmette-guérin; OPV, oral polio vaccine; pentavalent, diphtheria, tetanus, and pertussis (DTaP-3), hib and hepatitis B; PCV, pneumococcal conjugate vaccine; IPV, inactivated polio vaccine; MCV, measles-containing vaccine.

* The SHRUCs survey did not ask about IPV2 or Typhoid and neither were implemented country-wide at the time the children in this survey were 9 months old.

**Table 2:** Survey targets, coverage, and demographic characteristics.

**Districts**	**Number of SHRUCs**	**Clusters**			**Households**				**Children 12**–**23 months**			**Parental education status (% literate**†**)**	
		**Sampled**	**Randomized**	**Surveyed**	**Target**	**Randomized**	**Completed**	**Response rate**	**N**	**Age in months (mean ± sd)**	**% male children**	**Mothers**	**Fathers**
**Overall**	**39**	**612**	**610**	**610**	**7,956**	**7,904**	**7,549**	**99.3 %**	**6,976**	**17.1 ± 3.4**	**53.5**	**27.0**	**36.9**
Peshawar	17	170	170	170	2,210	2,205	2,049	99.8 %	2,007	17.6 ± 3.4	51.1	32.7	52
Korangi	2	82	82	82	1,066	1,066	1,066	100 %	1,036	17.2 ± 3.5	53.9	44.7	54.7
Karachi East	1	45	45	45	585	585	585	100 %	571	17.0 ± 3.4	55.2	21.3	27
Karachi West	4	72	72	72	936	936	935	100 %	924	17.5 ± 3.5	53	39.1	48.5
Malir	1	18	18	18	234	234	234	100 %	226	16.8 ± 3.4	53.6	30.2	30.2
Killa Abdullah	5	90	90	90	1,170	1,170	1,163	100 %	896	15.8 ± 2.5	52.7	1.6	1.7
Pishin	3	45	43	43	585	538	466	92 %*	420	17.2 ± 3.3	55.7	11	20.4
Quetta	6	90	90	90	1,170	1,170	1,051	100 %	896	16.8 ± 3.2	56.2	9.2	22.1

† Having received one or more years of formal education

* Two PSUs in Pishin were dropped because they were commercial neighborhoods with no residents.

### 2.4 Data collection instruments and procedures

Structured questionnaires were used to obtain the survey data. The questionnaires were designed using WHO guidelines and manual 2018 for vaccination coverage cluster surveys [13] and modified according to the survey's objectives. Three questionnaires were used to collect the data: **1)** for house line listing; to collect household information about key demographic indicators to generate a sampling frame for the selection of target households, **2)** for household survey component; to collect basic demographic information on all usual household members, the household, and the dwelling, **3)** for immunization data of the eligible children; to assess immunization coverage in each targeted household.

All the questionnaires were translated into the local language (Urdu) and translated back to the English language and were also pilot field-tested in different non-targeted locations in the country.

The survey team was comprised of data collectors, team leaders, and district and provincial supervisors. Each data collection team consisted of three members: two female members and one male member. Before commencing field activities, team leaders and supervisors underwent comprehensive central training in Karachi. This five-day training program was conducted by experienced investigators and faculty members from Aga Khan University. Subsequently, additional cascading training sessions were held for the data collection teams at the divisional level.

### 2.5 Data processing and analysis

Data collection was carried out in two phases in each district. In the first phase, household line listing was conducted in the selected clusters. The household listing was then used to select 13 eligible households from each cluster. Phase two was dedicated to collecting information on household socioeconomic status and routine immunization of children aged between 12 and 23 months. The routine immunization schedule of Pakistan is presented in Table 1.

The data were collected using handheld devices having Android compatible custom-made data collection applications. The data stored in the handheld devices were transmitted to the data center using internet connectivity. Key immunization results were tabulated against wealth quintiles, child's sex, urban versus rural population proportion, and parental education.

The data analysis was undertaken using STATA version 17 [14]. Survey weights were calculated following Annex J of the WHO vaccination coverage cluster survey reference manual 2018 [13].

Because data are to be combined across UCs to estimate SHRUC coverage at the district level, the weights were post-stratified, so the sum of weights in each UC would be proportional to the estimated population of eligible children there. In addition, administrative estimates of the population of children under five years of age in each SHRUC were obtained from the polio program. For the calculation of post-stratified weights, the number of children aged 12–23 months in each SHRUC was assumed to be proportional to the number of children under five years.

Bivariate and multivariate logistic regression was run to check the relationship of various possible factors with variation in immunization coverage at the district level of the target SHRUCs. Full immunization was taken as the dependent variable. In contrast, several potential explanatory variables were explored, such as socio-demographic characteristics of mothers/caregivers (wealth quintiles and education level) and child's sex. Using bivariate analysis, full vaccination status was tested with each predictor. Then, a multivariate analysis was performed for the predictors. Variables with a p-value ≤ 0.25 in bivariate were retained in multivariate analysis. Further, in the multivariate model, variables at p-value = 0.05 were considered significant for analysis.

A child is considered fully vaccinated when there is evidence (by card or by recall) of having received Bacillus Calmette-Guérin (BCG), oral polio vaccine (OPV 0, 1–3), pentavalent 1–3, pneumococcal conjugate vaccine (PCV 1–3), inactivated polio vaccine (IPV), and measles-containing vaccine (MCV1 – given at nine months of age) as per the schedule of the EPI Pakistan (Table 1) [15]. This definition omits the ROTA virus vaccine (RV 1–2) because these doses were introduced into the EPI schedule most recently in 2017 [16]. If he/she has received some but not all of those doses, considered partially vaccinated. And if the child has received none of those doses, he/she is classified as not vaccinated. Data was not collected on IPV2 or Typhoid vaccine since these doses had not been implemented nationwide when the children surveyed were nine months old.

### 2.6 Ethical approvall

Ethical approval was obtained from the National Bioethics Committee (NBC) No.4-87/NBC-379/19/1996 and the Aga Khan University (AKU) Ethical Review Committee (ERC) 2019-0652-5699 to implement the proposed survey activities. In addition, no objection certificates and approvals were obtained from the provincial and district authorities with the support of the National Program Manager EPI, Ministry of National Health Services Regulation & Coordination (MoNHSRC), Islamabad, Pakistan.

## 3 Results

The survey covered 610 clusters from the target SHRUCs and interviews were completed for 7,549 households with a response rate of 99.3 %. Overall, 6,976 children in the age group 12–23 months with a mean age of 17.1 (SD ± 3.4) were covered; more than half (53.5 %) were male children. From the SHRUC districts, a total of 27 % of mothers and 36.9 % of fathers were found literate - having received one or more years of formal education. District-wise survey targets, and coverage, are summarized in Table 2.

### 3.1 Immunization coverage for polio vaccines

Table 3 presents the coverage for polio vaccines (OPV 0–3, and IPV) for each SHRUC district. We analyzed the data in three different combinations: children vaccinated for polio with at least three doses of OPV and one dose of IPV, children vaccinated with any OPV doses or IPV and a third combination is of children who did not receive any polio vaccines.

**Table 3:** Immunization coverage for polio vaccines.

**District**	**OPV0**	**OPV 1**	**OPV 2**	**OPV 3**	**IPV**	**Vaccinated with at least 3 OPV doses and 1 IPV dose**	**Vaccinated with any OPV doses or IPV dose**	**Not vaccinated with OPV and IPV**	**Total number of children**
**Overall**	**78.9**	**77.7**	**69.1**	**62.0**	**64.6**	**60.9**	**20.4**	**18.7**	**6,976**
Peshawar	93.5	94.0	84.2	77.3	89.1	80.7	14.7	4.6	2,007
Korangi	80.4	78.0	70.0	62.9	60.6	60.1	22.1	17.7	1,036
Karachi West	76.7	73.3	66.3	61.3	63.1	60.0	17.8	22.2	924
Karachi East	82.3	79.6	72.5	63.4	60.8	59.9	24.4	15.7	571
Malir	75.2	71.5	62.5	56.9	53.9	53.4	23.1	23.5	226
Killa Abdullah	54.7	66.1	51.8	43.4	48.5	44.2	23.8	32.0	896
Pishin	63.9	62.1	50.8	43.7	48.5	43.7	21.7	34.6	420
Quetta	64.8	61.2	54.3	46.5	45.5	41.9	24.1	33.9	896

Abbreviations: OPV, oral polio vaccine; pentavalent, diphtheria, tetanus, and pertussis (DTaP-3), hib and hepatitis B; IPV, inactivated polio vaccine.

The results reflected that, overall, 60.9 % of children from the SHRUC districts were vaccinated with at least three doses of OPV and one dose of IPV, while 20.4 % were vaccinated with any OPV doses or IPV and 18.7 % of children did not receive any polio vaccines.

Looking at individual districts, Peshawar recorded the highest proportion of children (80.7 %) vaccinated with at least three doses of OPV and one dose of IPV. However, in the remaining SHRUC districts, the coverage proportion was recorded below 60 % with the lowest coverage in district Quetta (41.9 %).

The proportion of children vaccinated with any OPV doses or IPV dose across the SHRUC districts showed that except for districts Peshawar and Karachi West, in the remaining districts, more than 20 % of children were vaccinated with any OPV doses or IPV, with height record in Karachi East at 24.4 %.

Regarding the third combination, for children who were not vaccinated with any of the polio vaccines, the lowest proportion was recorded in district Peshawar, while the highest proportion was recorded in district Pishin at 34.6 %.

### 3.2 Immunization coverage

Table 4 presents the proportion of fully, partially, and not-vaccinated children for each SHRUC district. A child is considered fully vaccinated when there is evidence (by card or by recall) of having received BCG, OPV0, OPV 1–3, Penta 1–3, PCV 1–3, IPV, and MCV1 as per the EPI schedule in Pkaistan (Table 1) [15]. If the child has received none of those doses, he/she is classified as not vaccinated. And if he/she has received some but not all of those doses, considered partially vaccinated.

**Table 4:** Immunization coverage in SHRUC districts.

**Districts**																**Fully immunized**	**Partially immunized**	**Not immunized**	**Total number of children**
	**BCG**	**OPV0**	**OPV 1**	**Penta 1**	**PCV 1**	**RV 1**	**OPV 2**	**Penta 2**	**PCV 2**	**RV 2**	**OPV 3**	**Penta 3**	**PCV 3**	**IPV**	**MCV 1**				
**Overall**	**81.2**	**78.9**	**77.7**	**74.4**	**73.7**	**73.7**	**69.1**	**66.6**	**66.1**	**65.6**	**62**	**58.4**	**56.9**	**64.6**	**59.4**	**48.3**	**35.4**	**16.3**	**6,976**
Peshawar	94	93.5	94	90.2	90	89.6	84.2	86.8	86.6	85.2	77.3	82.5	81.7	89.1	81.9	70.1	25.9	4.1	2,007
Korangi	83.7	80.4	78	75.5	75.5	75.4	70	66.9	66.5	66.3	62.9	57.7	56.6	60.6	56.4	47.8	37.6	14.7	1,036
Karachi East	85.8	82.3	79.6	75.9	75.5	75.5	72.5	67.2	66.6	65.9	63.4	57.9	57.3	60.8	55.5	47.2	39.5	13.3	571
Karachi West	78.5	76.7	73.3	72.3	71.9	70.7	66.3	64.6	63.8	62.9	61.3	57.2	53.2	63.1	56.8	46.9	33.0	20.1	924
Malir	76.6	75.2	71.5	68.9	68.1	69.4	62.5	59.3	59.3	58.8	56.9	47.1	45.4	53.9	49.4	39.7	38.5	21.7	226
Killa Abdullah	64.1	54.7	66.1	54.6	49.4	54.1	51.8	38.9	36.5	42.7	43.4	21.3	19.5	48.5	45.8	14.8	65.4	19.8	896
Pishin	64.8	63.9	62.1	62.1	62.1	59.8	50.8	49.8	49.8	44.3	43.7	43.7	43.3	48.5	43.9	35.0	30.9	34.1	420
Quetta	65.7	64.8	61.2	59.8	59.3	58.4	54.3	52.1	51.5	50.9	46.5	45.9	44.9	45.5	43.8	36.6	30.4	33.0	896

Abbreviations: BCG, bacillus calmette-guérin; OPV, oral polio vaccine; pentavalent, diphtheria, tetanus, and pertussis (DTaP-3), hib and hepatitis B; PCV, pneumococcal conjugate vaccine; IPV, inactivated polio vaccine; MCV, measles-containing vaccine.

The results reflected that district Peshawar recorded the highest proportion of fully vaccinated children at 70.1 % [CI: 66.5 – 73.4]. However, in the remaining SHRUC districts, the proportion of fully vaccinated children was recorded below 50 %. Comparatively, a low proportion of fully vaccinated children was recorded in district Killa Abdullah at 14.8 % [CI: 10.5 – 20.5].

### 3.3 Coverage by antigen

Table 4 and table S1 presents antigen-wise coverage in each SHRUC district. District Peshawar recorded the highest coverage for all vaccines, followed by the SHRUC districts in Sindh, whereas comparatively low coverage was recorded in the SHRUC districts of Balochistan. The coverage results reflected that in each district, the coverage rate for OPV, Penta, PCV, and RV decreased with each next vaccine dose, indicating dropout in routine immunization.

### 3.4 Dropout rates between dose pairs in SHRUC districts

Dropout between vaccination visits is a constant feature of routine immunization [15], [16], and this survey also observed the dropout between dose pairs in the target districts. A dropout rate greater than 10 % is considered a 'high dropout' by WHO as a global vaccination practice [17]. The dropout rates are relative percentages, ie., calculated using the formula [Dropout rate = ((earlier dose coverage - later dose coverage) /earlier dose coverage)*100].

Table 5 indicates that dropout was higher than 10 % for most of the dose series in most districts as measured by the SHRUCs survey. Drop-out was especially high in Killa Abdullah for all dose pairs. The estimates for Penta1 to Penta3 and PCV1 to PCV3 dropout were notably high in Killa Abdullah.

**Table 5:** Drop-out rates between dose pairs in SHRUC districts.

**Districts**	**OPV0 - OPV3 dropout (%)**	**OPV1 - OPV3 dropout (%)**	**Penta1 - Penta3 dropout (%)**	**PCV1 - PCV3 Dropout (%)**	**RV1 - RV2 dropout (%)**	**BCG - MCV1 dropout (%)**	**Penta1 - MCV1 dropout (%)**
Karachi West	20.1	16.4	20.8	25.9	11.0	27.7	21.4
Peshawar	17.4	17.8	8.5	9.2	4.9	12.9	9.2
Korangi	21.8	19.4	23.6	25.0	12.0	32.6	25.3
Karachi East	23.0	20.4	23.8	24.1	12.7	35.3	26.9
Malir	24.4	20.5	31.6	33.3	15.2	35.5	28.3
Quetta	28.3	24.1	23.3	24.2	13.0	33.3	26.7
Pishin	31.6	29.6	29.6	30.2	26.0	32.3	29.3
Killa Abdullah	20.6	34.3	61.0	60.5	21.2	28.5	16.1

Abbreviations: BCG, bacillus calmette-guérin; OPV, oral polio vaccine; pentavalent, diphtheria, tetanus, and pertussis (DTaP-3), hib and hepatitis B; PCV, pneumococcal conjugate vaccine; IPV, inactivated polio vaccine; MCV, measles-containing vaccine.

For polio vaccines, there were significant dropout rates observed between OPV0 and OPV3 in districts of Karachi and Quetta, with Pishin recording an the SHRUCshigher rate of 31.6 %. A similar trend in dropout rates between OPV1 and OPV3 was noted in the SHRUCs districts, particularly higher in Killa Abdullah. Notably, the dropout rates for the polio vaccines, both between OPV0 and OPV3 and between OPV1 and OPV3, were higher in the district Peshawar compared to other vaccine dose pairs.

The dropout rates between the first and second doses of the Rotavirrus vaccine (RV) vaccine were the lowest for all SHRUC districts as compared to other vaccine dose pairs. Even though, for all districts except district Peshawar, the RV dropout was higher than 10 %. On the other hand, the dropout rates between BCG and MCV1 were higher than other dose pairs for all SHRUC districts except district Killa Abdullah. The dropout between Penta1 and MCV1 also depicted higher rates, except in district Peshawar. Interestingly, the dropout for this particular dose is lowest in Killa Abdullah when compared to other vaccine dose pairs in the district.

### 3.5 Determinants of vaccination status

The multivariate regression results **(**Table 6**)** reflected that maternal education; primary [OR 0.71; 95 % CI 0.50 – 1.00], higher education (11 years and above) [OR 1.54; 95 % CI 1.04 – 2.26], paternal education; middle [OR 1.37; 95 % CI 1.03 – 1.81], secondary [OR 1.75; 95 % CI 1.39 – 2.19], and higher (11 years and above) [OR 1.66; 95 % CI 1.27 – 2.17] compared to no education was more likely to be associated with full vaccination of the children. And full vaccination was found significant among the children of any SHRUC district compared to district Killa Abdullah.

**Table 6:** Factors associated with full immunization status of children aged 12–23 months in SHRUC district- A logistic regression analysis.

	**Bivariate**				**Multivariate**			
	**OR**	**95 % CI**		**P-value**	**OR**	**95 % CI**		**P-value**
**Child sex**								
Male	Ref.							
Female	1.07	0.94	1.21	0.294				
**Mother education**								
None	Ref.				Ref.			
Primary (1–5)	0.93	0.61	1.41	0.726	0.71	**0.50**	**1.00**	0.048
Middle (6–8)	1.48	1.10	1.99	0.010	0.91	0.65	1.25	0.552
Secondary (9–10)	2.04	1.60	2.59	0.000	1.21	0.93	1.59	0.162
Higher (11 and above)	2.91	2.13	3.96	0.000	1.54	**1.04**	**2.26**	0.029
**Father education**								
None	Ref.				Ref.			
Primary (1–5)	1.04	0.68	1.58	0.870	1.03	0.74	1.44	0.847
Middle (6–8)	1.78	1.37	2.32	0.000	1.37	**1.03**	**1.81**	0.031
Secondary (9–10)	2.26	1.83	2.79	0.000	1.75	**1.39**	**2.19**	0.000
Higher (11 and above)	2.90	2.31	3.63	0.000	1.66	**1.27**	**2.17**	0.000
**District**								
Killa Abdullah	Ref.				Ref.			
Peshawar	13.50	8.79	20.73	0.000	10.72	**6.98**	**16.47**	0.000
Korangi	5.27	3.42	8.13	0.000	4.27	**2.75**	**6.63**	0.000
Karachi East	5.16	3.20	8.30	0.000	4.80	**2.96**	**7.77**	0.000
Karachi West	5.10	3.23	8.05	0.000	4.17	**2.64**	**6.59**	0.000
Malir	3.80	2.10	6.90	0.000	3.60	**2.07**	**6.24**	0.000
Pishin	3.11	1.81	5.34	0.000	2.77	**1.65**	**4.64**	0.000
Quetta	3.33	2.15	5.18	0.000	3.00	**1.94**	**4.65**	0.000

### 3.6 Vaccination home-based record (card) availability

Three-quarters or more of the children in Sindh and KP SHRUC districts reported to have received a vaccination card, whereas only one- to two-thirds of the children in the Balochistan SHRUCs did so (Table 7).

**Table 7:** Vaccination card availability, reasons for non-availability of vaccination cards and not vaccinating children.

		**Peshawar**	**Korangi**	**Karachi East**	**Karachi West**	**Malir**	**Killa Abdullah**	**Pishin**	**Quetta**
**Ever had a vaccination card**		91.1	81.7	81	75.4	73.8	34.3	66.9	67.5
**Card retention (seen by the survey interviewer)**		73.5	58.8	52.1	51.8	42.5	18.1	28.3	30.2
Reasons for never having received a vaccination card*	Don’t think it’s important	2.4	7.4	10.1	10	3.2	38.3	18.9	6.2
	Never visited a facility	2.7	8.2	7.3	8.7	19.2	2.4	13	25.5
	Card was not available with the health provider	3	0.2	0.3	0	1.5	0.5	0.2	0.3
	The vaccinator/ facility didn’t provide the card	0.3	0	0	0.2	0	0.5	0	0.1
	Not aware of such cards	0	0.3	0.3	0.4	0	18.8	0.2	0.2
	Other specify	0.5	2.3	0.9	5.4	2.2	5.1	0.9	0.1
**Reasons for not showing a vaccination card**^	Card not found at this time	7	7.3	8.9	6.1	10	7.7	12.3	12.2
	Card misplaced	8.6	13.1	15.6	13.5	18.2	6.8	20.3	23.2
	Card is with the vaccinator	1.4	0.3	2.2	0.7	2.3	1.7	4	0.8
	Other	0.5	2.2	2.2	3.2	0.9	0.1	2	1.1
**Reasons for not vaccinating children**†	Place of immunization too far	0.0	0.5	0.9	1.6	1.3	0.3	2.9	2.6
	Time of immunization not convenient	0.0	2.7	0.7	0.3	4.9	0.0	0.9	0.3
	Mother too busy	0.1	1.1	1.3	1.1	2.2	0.0	2.9	1.6
	Family problem including mother ill	0.2	1.0	1.7	0.5	1.8	0.0	1.9	4.1
	Child ill, not brought	0.4	1.8	2.3	3.2	5.4	0.0	3.1	4.6
	Child ill, brought but not vaccinated	0.0	0.2	1.0	0.1	0.8	0.1	0.8	0.4
	Long wait	0.0	0.0	0.0	0.0	0.4	0.6	1.2	0.8
	Rumors	1.2	3.2	4.7	5.7	5.8	9.4	2.5	3.6
	No faith in immunization	1.7	3.7	5.2	5.7	7.6	6.3	10.0	9.0
	Fear of side reaction	1.1	4.0	2.3	4.6	5.9	3.0	7.1	2.9
	Time or Place of immunization not Known	0.0	0.3	0.0	0.2	0.4	0.2	2.7	1.7
	Took child but no vaccine available	0.1	0.0	0.4	0.0	0.0	0.0	0.0	0.0
	Took child but no vaccinator	0.0	0.1	0.0	0.0	1.0	0.1	0.0	0.0
	Took child facility closed	0.0	0.0	0.0	0.0	0.0	0.0	0.2	0.0
	Took child but not vaccination day	0.0	0.0	0.0	0.1	0.0	0.1	0.7	0.0
	Family does not allow	0.0	2.1	2.2	1.6	0.9	0.0	0.0	0.0
	Other	0.1	0.2	0.0	0.0	0.0	0.0	2.1	0.1

* Each column sums to the % of children who never received a card.

^ Each column sums to the % of children who haven't shown the card.

† Each column sums to the % of children who are not immunized at all.

For every SHRUC district, a substantial portion of caregivers who reported having received a vaccination card for the child were unable to show it to the surveyor. In district Peshawar for a higher percentage (73.5 %) of children cards were seen during the survey. In contrast, district Killa Abdullah had the lowest proportion (18.1 %) of cards seen by the survey team at the time of the household visit.

### 3.7 Reason for not vaccination and non-availability of vaccination card

The primary reasons for not vaccinating children in the target districts were rumors, lack of faith in immunization, and fear of the side effects of vaccines. Adding to these, a significant proportion of responses reflected that the place of immunization was too far, and mothers' busy schedules at the time of vaccination left the child unvaccinated.

In the target districts, a primary reason for the non-availability of vaccination cards was unawareness of the importance of the card. Another important reason was that family members of the children never visited a health facility to obtain a vaccination card. And in some of the districts, the family members were unaware of the vaccination card (Table 7**).**

## 4 Discussion

Since this survey was conducted at the union council level, the coverage results were aggregated at the district level. The SHRUCs constituted a subset of these districts, so the SHRUCs results are not meant to represent the entire district. However, the SHRUCs results aggregated at the district level reflected that overall full immunization coverage in the SHRUC districts was below the national FIC (66 %) in 2018 and 76.5 % in 2021 [4], [5]. A similar trend was reported by a study in polio-high districts of Northern Nigeria [18]. Compared with neighboring India, the FIC in the SHRUC districts, except districts of Balochistan, is comparatively higher than the low-performing district Mewat (38 %) in the Haryana state of India [19]. To add further, in the PDHS survey, the immunization coverage was typically the lowest for Balochistan. Consistent with these findings, this survey found that coverage of all routine antigens was lowest in the SHRUC districts of Balochistan.

Our study revealed that 60.9 % of children from the SHRUC districts received a minimum of three doses of OPV and one dose of IPV. Additionally, 20.4 % were vaccinated with any OPV doses or IPV and 18.7 % of children did not receive any polio vaccines. The results of our study on polio vaccination are comparable with similar studies. A 2016 study in Pakistan, based on a nationally representative sample, reported that 56.4 % of children aged up to 5 years had complete polio vaccination (all four oral polio vaccine (OPV) doses), 33 % had incomplete vaccination, and 10.3 % had no OPV doses [20]. Another study analyzed the polio vaccine coverage trend in Brazil and indicated that 70.1 % of children under one year of age had received three IPV doses and two OPV boosters [21]. Similarly, a study in Ethiopia showed that 72.6 % of children aged 12–23 months were fully vaccinated with polio vaccines in the CORE Group Polio Project (CGPP) area in 2017. In this study, full vaccination for polio was defined as receiving all three doses of OPV (OPV 1, OPV 2, and OPV 3) irrespective of whether the birth dose (OPV 0) had been administered [22].

Dropout between vaccination visits is common in routine immunization [17], [18]. In this survey, the dropout rates between BCG and MCV1, OPV0 and OPV3, and between OPV1 and OPV3 were substantially higher in all SHRUC districts than the WHO recommended cutoff point of 10 % [19]. In addition, dropout rates between Penta1 and Penta3, PCV1 and PCV3, and between RV1 and RV2 were higher than 10 % for all SHRUC districts except district Peshawar (Table 3). Earlier studies reported similar trends of dropout rates in routine immunization in Pakistan [23], in Low and Middle-Income Countries (LMICs) [24], [25], and in West Cameroon [26].

In the multivariate analysis, maternal and paternal education were significant determinants of full immunization among children aged between 12 and 23 months. The findings were consistent with previous studies in Sindh Pakistan [27], Afghanistan [28], East Africa [29], and India [30]. In addition, our findings reflected that compared to district Killa Abdullah, the full vaccination in other SHRUC districts was higher and statistically significant. Furthermore, no difference was found in full immunization based on the sex of a child.

The primary reasons reported for not vaccinating children were rumors about vaccines, lack of faith in immunization, fear of side effects of vaccines, poor access to vaccination sites, and mothers/care being too busy. Similar findings have been reported from community-based studies in Pakistan [27], [31], Afghanistan [28], and other LMICs in Africa [29], [32].

Since this survey reported the lowest card retention in the SHRUC districts of Balochistan, which is consistent with the PDHS survey findings [4], previous studies have reported low retention of vaccination cards in other LMICs; ranging from 20.7 % in the Democratic Republic of Congo [33] to 45 % in high-risk districts of Nigeria [34] and 69.2 % in South Africa [33]. An earlier study in Pakistan reported several reasons for not retaining a vaccination card, including large household size and the number of children in the household, gender of the child, mother/caregiver education, and access to mass media [35]. Contrary to that, a study from Uganda established that factors like mother's delivery in the health facility and usage of health services for antenatal care were associated with improved card retention rates [36].

Our survey findings showed that the lack of awareness regarding the importance of immunization among households and the timely unavailability of the vaccination card added to a low retention rate. Mostly, the mothers/caregivers never visited a facility to get a card and lacked awareness about the card and its importance. Thus, there is a need to sensitize the mothers/caregivers regarding the importance of keeping a vaccination card.

### 4.1 Strengths and limitations of the study

The strength of this survey lies in utilizing home-based record (HBR) photographs for the household that shows HBRs, to verify the recorded vaccination dates. The SHRUCs survey is the first in a series of surveys to monitor vaccination coverage in these critical UCs. Nonetheless, the survey had some limitations. Of the targeted 40 SHRUCs, one SHRUC was dropped from the survey scope due to security concerns. Furthermore, the survey dropped two PSUs in district Pishin because they were commercial neighborhoods with no residents.

## 5 Conclusion

The survey findings suggest that substantial efforts are needed to develop context-specific strategies and align them sustainably in the long run to maximize immunization coverage. On the demand side, more focus is required on active community engagement and targeted mobilization to bring about changes in perception regarding the importance and acceptance of vaccination. Coupled with these measures, increasing focus on supervision, and monitoring of immunization coverage activities, at the local level would help to improve immunization coverage in these areas.

## Author Contributions

SBS conceptualized and designed the survey and assisted with interpreting the results. IH developed the sampling strategy and tools and supervised the field activities. AK drafted the manuscript. DAR and CC performed the statistical analysis. IA and UA managed the data and performed the statistical analysis. MU assisted with study design, formulating contextually relevant survey tools, and oversaw data collection activity, and RMS reviewed the manuscript. All authors reviewed and approved the final manuscript for submission.

## Declaration of competing interest

The authors declare that they have no known competing financial interests or personal relationships that could have appeared to influence the work reported in this paper.

## Data Availability

Data will be made available on request.
