# Supplementary material for: Determinants of immunization in polio super high-risk union councils of Pakistan
Source: Vaccine. 2024 Jan 25;42(3):583–90. doi: 10.1016/j.vaccine.2023.12.056 (PMC10850981; doi:10.1016/j.vaccine.2023.12.056)
Supplement: Supplementary data 1 [file mmc1.docx]

**Determinants of Immunization in Polio Super High-Risk Union Councils of Pakistan**

**Figure S1. Map of Pakistan showing the super high risk union councils (SHRUCs) districts**


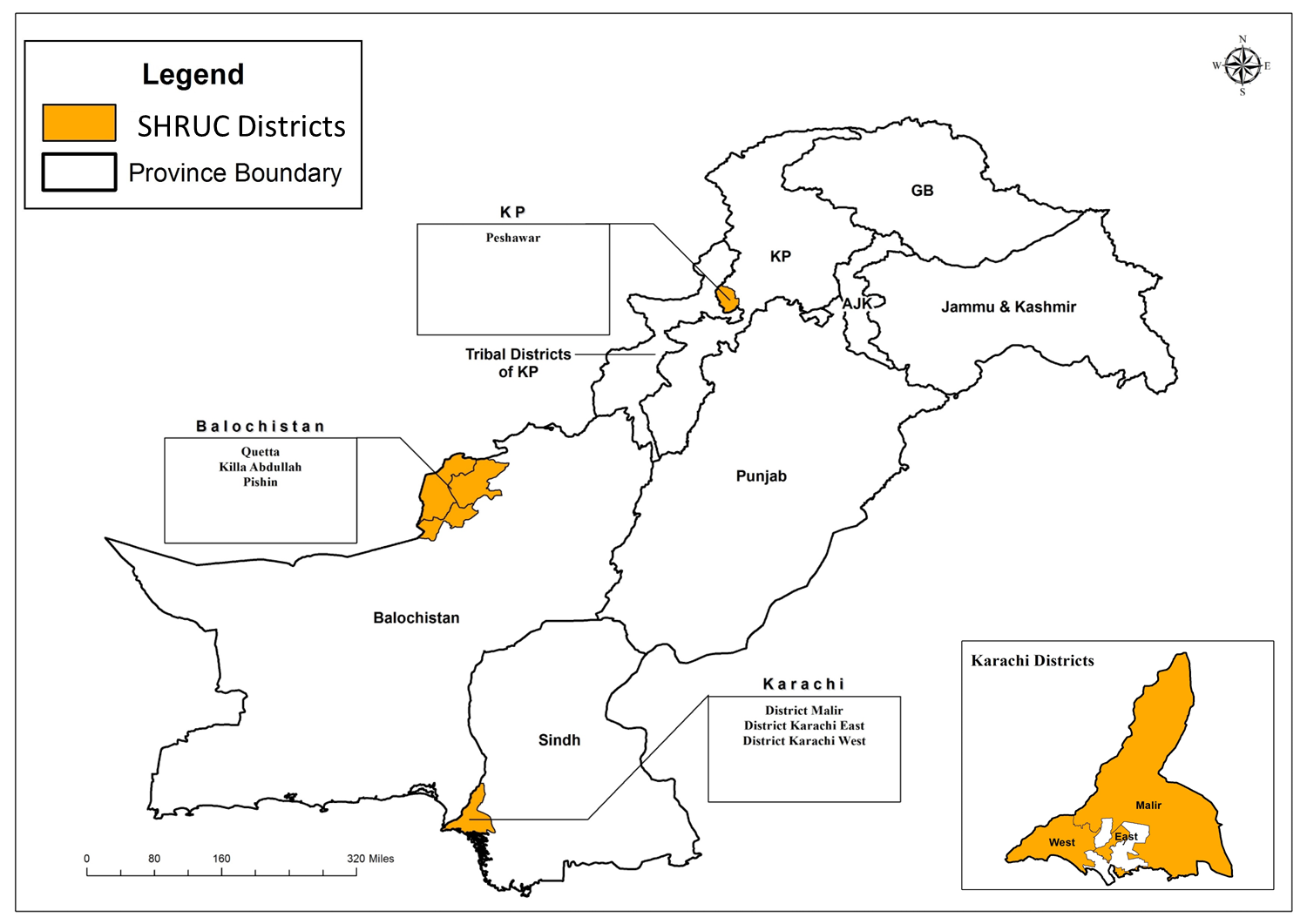


**Table S1. Immunization coverage with confidence intervals (CIs) in SHRUC districts**

| **District** | **BCG** | **OPV0** | **OPV 1** | **Penta 1** | **PCV 1** | **RV 1** | **OPV 2** | **Penta 2** | **PCV 2** | **RV 2** | **OPV 3** | **Penta 3** | **PCV 3** | **IPV** | **MCV 1** | **Fully immunized** | **Partially immunized** | **Not immunized** | **Total number of children** |
| --- | --- | --- | --- | --- | --- | --- | --- | --- | --- | --- | --- | --- | --- | --- | --- | --- | --- | --- | --- |
| **Overall** | 81.2  (79 - 83.3) | 78.9  (76.7 - 81.1) | 77.7  (75.5 - 79.8) | 74.4  (72.2 - 76.6) | 73.7  (71.5 - 75.9) | 73.7  (71.5 - 75.9) | 69.1  (66.9 - 71.3) | 66.6  (64.3 - 69) | 66.1  (63.7 - 68.4) | 65.6  (63.3 - 68) | 62.0  (59.7 - 64.2) | 58.4  (55.8 - 61) | 56.9  (54.2 - 59.5) | 64.6  (62.1 - 67.1) | 59.4  (57 - 61.8) | 48.3  (45.7 - 50.8) | 35.4  (33.3 - 37.5) | 16.3  (14.2 - 18.4) | 6,976 |
| Peshawar | 94.0  (92.3 - 95.7) | 93.5  (91.7 - 95.4) | 94.0  (92.4 - 95.6) | 90.2  (88.3 - 92.1) | 90.0  (88.2 - 91.9) | 89.6  (87.8 - 91.4) | 84.2  (81.9 - 86.4) | 86.8  (84.6 - 89) | 86.6  (84.4 - 88.9) | 85.2  (83 - 87.4) | 77.3  (74.1 - 80.4) | 82.5  (80.1 - 85) | 81.7  (79.3 - 84.2) | 89.1  (86.9 - 91.3) | 81.9  (79.2 - 84.5) | 70.1  (66.5 - 73.6) | 25.9  (22.8 - 29) | 4.1  (2.6 - 5.6) | 2,007 |
| Korangi | 83.7  (80.2 - 87.3) | 80.4  (76.7 - 84.2) | 78.0  (74 - 82) | 75.5  (71.6 - 79.5) | 75.5  (71.5 - 79.4) | 75.4  (71.3 - 79.4) | 70.0  (65.9 - 74.1) | 66.9  (62.7 - 71.2) | 66.5  (62.2 - 70.7) | 66.3  (62.1 - 70.6) | 62.9  (58.3 - 67.4) | 57.7  (53 - 62.5) | 56.6  (51.8 - 61.5) | 60.6  (56.1 - 65.1) | 56.4  (52.5 - 60.3) | 47.8  (43.3 - 52.3) | 37.6  (33.3 - 41.7) | 14.7  (11.2 - 18.2) | 1,036 |
| Karachi East | 85.8  (80.9 - 90.7) | 82.3  (76.9 - 87.7) | 79.6  (74.4 - 84.7) | 75.9  (70.3 - 81.5) | 75.5  (69.9 - 81.1) | 75.5  (69.9 - 81.2) | 72.5  (67.1 - 77.9) | 67.2  (61.6 - 72.8) | 66.6  (60.8 - 72.4) | 65.9  (60.1 - 71.7) | 63.4  (57.5 - 69.2) | 57.9  (51.5 - 64.3) | 57.3  (50.7 - 64) | 60.8  (54.4 - 67.2) | 55.5  (49.1 - 61.9) | 47.2  (40.3 - 54.1) | 39.5  (33.3 - 45.8) | 13.3  (8.4 - 18.1) | 571 |
| Karachi West | 78.5  (72.5 - 84.5) | 76.7  (70.9 - 82.5) | 73.3  (67.2 - 79.4) | 72.3  (66 - 78.5) | 71.9  (65.7 - 78) | 70.7  (64.7 - 76.7) | 66.3  (60.5 - 72.2) | 64.6  (58.6 - 70.7) | 63.8  (57.8 - 69.8) | 62.9  (56.9 - 68.9) | 61.3  (55.1 - 67.5) | 57.2  (50.8 - 63.6) | 53.2  (47.1 - 59.4) | 63.1  (56.7 - 69.5) | 56.8  (50.8 - 62.7) | 46.9  (41.1 - 52.7) | 33.0  (28.3 - 37.8) | 20.1  (14 - 26.1) | 924 |
| Malir | 76.6  (64.5 - 88.6) | 75.2  (63.7 - 86.7) | 71.5  (61.2 - 81.9) | 68.9  (58.7 - 79.1) | 68.1  (57.7 - 78.4) | 69.4  (58.9 - 79.8) | 62.5  (52.5 - 72.6) | 59.3  (48.6 - 70) | 59.3  (48.6 - 70) | 58.8  (47.9 - 69.7) | 56.9  (47.4 - 66.3) | 47.1  (34.3 - 59.9) | 45.4  (32 - 58.7) | 53.9  (43.9 - 63.8) | 49.4  (40.2 - 58.6) | 39.7  (28 - 51.5) | 38.5  (28.4 - 48.6) | 21.7  (10.4 - 33.1) | 226 |
| Killa Abdullah | 64.1  (56.3 - 72) | 54.7  (47.8 - 61.7) | 66.1  (58.8 - 73.4) | 54.6  (47.8 - 61.4) | 49.4  (42.4 - 56.4) | 54.1  (46.1 - 62.1) | 51.8  (43 - 60.6) | 38.9  (31.2 - 46.6) | 36.5  (29 - 44) | 42.7  (33.9 - 51.4) | 43.4  (35.1 - 51.8) | 21.3  (14.9 - 27.7) | 19.5  (13.4 - 25.6) | 48.5  (39.3 - 57.7) | 45.8  (38.7 - 52.9) | 14.8  (9.7 - 19.9) | 65.4  (59.5 - 71) | 19.8  (14.8 - 25) | 896 |
| Pishin | 64.8  (58.1 - 71.5) | 63.9  (57.2 - 70.6) | 62.1  (55.2 - 69) | 62.1  (55.4 - 68.8) | 62.1  (55.4 - 68.8) | 59.8  (53.4 - 66.3) | 50.8  (42.9 - 58.6) | 49.8  (41.4 - 58.3) | 49.8  (41.4 - 58.3) | 44.3  (36 - 52.6) | 43.7  (35.4 - 52) | 43.7  (34.5 - 52.9) | 43.3  (34.1 - 52.6) | 48.5  (40.4 - 56.5) | 43.9  (35.5 - 52.3) | 35.0  (25.9 - 43.6) | 30.9  (24.1 - 37.3) | 34.1  (28 - 41.2) | 420 |
| Quetta | 65.7  (61.2 - 70.2) | 64.8  (60.3 - 69.4) | 61.2  (56.5 - 66) | 59.8  (55.1 - 64.5) | 59.3  (54.5 - 64) | 58.4  (53.6 - 63.3) | 54.3  (49.4 - 59.1) | 52.1  (47.2 - 56.9) | 51.5  (46.5 - 56.4) | 50.9  (45.8 - 55.9) | 46.5  (42.1 - 50.9) | 45.9  (41.5 - 50.3) | 44.9  (40.5 - 49.3) | 45.5  (40.7 - 50.2) | 43.8  (39.1 - 48.6) | 36.6  (31.9 - 41.1) | 30.4  (26.4 - 34.4) | 33.0  (28.6 - 37.5) | 896 |
